# Arginase Activity in the Blood of Patients with Visceral Leishmaniasis and HIV Infection

**DOI:** 10.1371/journal.pntd.0001977

**Published:** 2013-01-17

**Authors:** Yegnasew Takele, Tamrat Abebe, Teklu Weldegebreal, Asrat Hailu, Workagegnehu Hailu, Zewdu Hurissa, Jemal Ali, Ermiyas Diro, Yifru Sisay, Tom Cloke, Manuel Modolell, Markus Munder, Fabienne Tacchini-Cottier, Ingrid Müller, Pascale Kropf

**Affiliations:** 1 Gondar University Leishmaniasis Research and Treatment Centre, University of Gondar, Gondar, Ethiopia; 2 Department of Microbiology, Immunology, Parasitology and Pathology, Addis Ababa University, Addis Ababa, Ethiopia; 3 College of Medicine and Health Sciences, University of Gondar, Gondar, Ethiopia; 4 Department of Microbiology, Immunology and Parasitology, School of Biomedical and Laboratory Sciences, University of Gondar, Gondar, Ethiopia; 5 Department of Medicine, Section of Immunology, Imperial College London, London, United Kingdom; 6 Department of Cellular Immunology, Max-Planck-Institute for Immunobiology and Epigenetics, Freiburg, Germany; 7 Third Department of Medicine (Hematology, Oncology, and Pneumology), University Medical Center Mainz, Mainz, Germany; 8 Department of Biochemistry, WHO Immunology Research and Training Center, University of Lausanne, Lausanne, Switzerland; 9 Department of Immunology and Infection, London School of Hygiene and Tropical Medicine, London, United Kingdom; Institut Pasteur de Tunis, Tunisia

## Abstract

**Background:**

Visceral leishmaniasis is a parasitic disease associated with high mortality. The most important foci of visceral leishmaniasis in Ethiopia are in the Northwest and are predominantly associated with high rates of HIV co-infection. Co-infection of visceral leishmaniasis patients with HIV results in higher mortality, treatment failure and relapse. We have previously shown that arginase, an enzyme associated with immunosuppression, was increased in patients with visceral leishmaniasis and in HIV seropositive patients; further our results showed that high arginase activity is a marker of disease severity. Here, we tested the hypothesis that increased arginase activities associated with visceral leishmaniasis and HIV infections synergize in patients co-infected with both pathogens.

**Methodology/Principal Findings:**

We recruited a cohort of patients with visceral leishmaniasis and a cohort of patients with visceral leishmaniasis and HIV infection from Gondar, Northwest Ethiopia, and recorded and compared their clinical data. Further, we measured the levels of arginase activity in the blood of these patients and identified the phenotype of arginase-expressing cells. Our results show that CD4^+^ T cell counts were significantly lower and the parasite load in the spleen was significantly higher in co-infected patients. Moreover, our results demonstrate that arginase activity was significantly higher in peripheral blood mononuclear cells and plasma of co-infected patients. Finally, we identified the cells-expressing arginase in the PBMCs as low-density granulocytes.

**Conclusion:**

Our results suggest that increased arginase might contribute to the poor disease outcome characteristic of patients with visceral leishmaniasis and HIV co-infection.

## Author Summary

Visceral leishmaniasis (VL) in Ethiopia is caused by the parasite *Leishmania donovani*. This disease has one of the highest mortality rates: if left untreated, it is almost always fatal. VL belongs to the most neglected tropical diseases, affecting the poorest populations, for whom access to diagnosis and effective treatment are often not available. VL is worsened when the patients are co-infected with HIV. We have recently shown that patients with visceral leishmaniasis (VL patients) and HIV seropositive patients (HIV+ patients) have increased levels of an enzyme, arginase, in their blood. This enzyme has been shown to prevent cells of the immune system from mounting an efficient response and controlling the replication of the virus in HIV+ patients or the parasites in VL patients. In this study, we show that arginase is considerably higher in the blood of VL/HIV co-infected patients as compared to VL patients. Our results suggest that this abnormally high arginase might contribute to the poor prognosis associated to VL/HIV patients.

## Introduction

Visceral leishmaniasis (VL), a neglected tropical disease caused by the parasite *Leishmania (L.) donovani*, is one of the most significant vector-borne diseases in Ethiopia, with an estimated 4500 to 5000 new cases every year [2]. VL is associated with high mortality and morbidity and hinders economic development. VL is worsened by malnutrition and co-infections with HIV. In the region with the highest endemicity for VL in Northwest Ethiopia, the prevalence of HIV seropositivity among patients with VL has been shown to be as high as 38% [1]. Patients with visceral leishmaniasis and HIV infection (VL/HIV patients) display an increased susceptibility to drug toxicity, increased leishmaniasis relapses and increased mortality [1], [3], [4]; and accelerated progression to AIDS [5].

Both *Leishmania* and HIV are able to infect and replicate in monocytes and macrophages and both pathogens mutually promote their replication in these host cells. Several studies have shown that infection of myeloid cells with *Leishmania* parasites promotes HIV replication [6], [7], [8]. Equally, HIV not only promotes *Leishmania* uptake by macrophages [9], but also increases parasite replication in monocytes [10]; this is in agreement with the observation of increased parasitemia in VL/HIV patients [11].

One of the immunological hallmarks of VL and HIV infections is a diminished ability of PBMCs from these patients to respond to recall antigens [12], [13], [14]. We have recently shown that the activity of the enzyme arginase is increased in patients with visceral leishmaniasis (VL patients) and in HIV seropositive patients (HIV patients) with low CD4^+^ T cell counts [15], [16]. Arginase hydrolyzes L-arginine to urea and ornithine, which is further metabolized into polyamines. Arginase can also be upregulated in myeloid cells and has been shown to impair T cell responses by reducing the bioavailability of L-arginine in the microenvironment. Since L-arginine is essential for efficient T cell activation, this decrease results in impaired T cell responses [17], [18], [19]. In both our HIV [20] and VL [16] studies, increased arginase activity in PBMCs was a marker of disease severity, and coincided with lower L-arginine levels and impaired T cell responses.

Here, we tested the hypothesis that a synergistic increase in arginase activity occurs in VL patients co-infected with HIV as compared to VL patients, and therefore contributes to exacerbated disease outcomes.

## Materials and Methods

### Subjects and sample collection

The study was approved by the IRB of College of Medicine and Health Science, University of Gondar, reference number 09/07/2003. For this cross-sectional study, a cohort of 26 patients with visceral leishmaniasis, but HIV seronegative uninfected (VL patients) and 14 VL/HIV co-infected patients was recruited from the Leishmaniasis Treatment and Research Center of Gondar University Hospital; informed written consent was obtained from each patient. All patients recruited in our study were migrant workers and male.

The diagnosis of VL was based on positive serology (rK39, DiaMed IT Leish, DiaMed AG, Cressiers/Morat, Switzerland) and presence of amastigotes in spleen or bone marrow aspirates. HIV seropositivity was based on the following tests: KHB Shanghai Kehua Bio-engineering Co. Ltd and Chembio HIV 1/2 STAT-PAK; Uni-Gold (Trinity Biotech PLC) was used to resolve ambiguous results. Out of the 14 co-infected patients, 10 were primary VL patients and 4 had a relapse of VL. Relapse is defined as follows: individuals diagnosed with visceral leishmaniasis (clinical features and positive parasitology), after having been successfully treated for primary VL and been discharged with an improved medical condition or with a negative test of cure. Six patients were already on anti-retroviral therapy (ART), the 8 remaining patients were initiated on ART after the treatment against VL. The treatment was given according to the recommendation of the National Guideline for Diagnosis, Treatment & Prevention of Leishmaniasis in Ethiopia: VL patients HIV+ already on ART were treated with AmBisome (Gilaed Sciences Ltd. (Ireland)) at a dose of 3–5 mg/kg daily or intermittently for 10 doses, up to a total dose of 40 mg/kg. VL patients HIV+ not on ART were treated with SSG (20 mg/kg for 30 days) first and started ART upon completion of the treatment against VL.

All the VL patients were treated with SSG for 30 days at a dose of 20 mg/kg.

Informed written consent was obtained from each patient. The median age of the VL patients was 22.0±1.0 years (range: 16–32 years) and that of VL/HIV co-infected patients was 33.0±1.9 years (range: 28–38 years).

Saliva was collected by spitting in a sterile 50 ml tube, after the patients rinsed their mouth with clean water. The saliva sample was immediately frozen at −20°C.

Ten to twenty ml of venous blood was collected into EDTA tubes before the antileishmanial treatment started. Plasma was isolated by centrifuging 1 ml of blood at 1800 rpm for 10 minutes and was frozen at −20°C until further use. Peripheral blood mononuclear cells (PBMCs) were isolated by density gradient centrifugation on Histopaque-1077 (Sigma). Cells were washed in phosphate buffered saline (PBS) and used straight away for flow cytometry; PBMCs used for arginase and protein determination were immediately resuspended in lysis buffer (0.1% Triton X-100, 25 mM Tris-HCl and 10 mM MnCl_2_, Sigma) and frozen at −20°C until further use.

### Laboratory evaluation

CD4^+^ and CD8^+^ T cell counts were determined using a BD Multi TEST kit (BD Biosciences) and acquisition was performed using a FACSCalibur (BD Biosciences).

Liver and renal function tests were performed as part of the routine management practice. The following reagents (HUMAN Diagnostics) were used according to the manufacturer's protocol: GOT (ASAT); Aspartate aminotransferase liquiUV test; GPT (ALAT), Alanine aminotransferase liquiUV test; ALP, Alkaline Phosphatase liquicolor test; BUN (Blood Urea Nitrogen), Urea liquicolor test; Creatinine, creatinine liquicolor test.

Determination of parasite burden: grading was assessed as previously described in the splenic aspirates [21] and the same grading system was applied for the bone marrow aspirates.

### Determination of arginase activity

The enzymatic activity of arginase was measured as previously described [15]. To activate arginase, 50 µl of PBMCs (resuspended in lysis buffer = 0.1% Triton X-100/10 mM MnCl_2_/25 mM Tris-HCl) were incubated for 10 min at 56°C. Arginine hydrolysis was achieved by incubating the lysate with 50 µl of 0.5 M L-arginine (pH 9.7) at 37°C for 15–120 min. The reaction was stopped with 400 µl of H_2_SO_4_ (96%)/H_3_PO_4_ (85%)/H_2_O (1/3/7, v/v/v). Urea concentration was measured at 550 nm after addition of 20 µl α-isonitrosopropiophenone (dissolved in 100% ethanol), followed by heating at 100°C for 45 min.

To determine arginase activity in the plasma, urea concentrations were first determined in the sera, without the activation and hydrolysis steps; these values were subtracted from those obtained by measuring the urea levels as described above.

One unit of enzyme activity is defined as the amount of enzyme that catalyzes the formation of 1 µmol of urea per min.

### Protein determination

To determine the protein concentration of lysed PBMC samples, serial dilutions of each PBMC lysate were made in PBS (Sigma). BCA Protein Assay Reagent (Pierce) was added to each PBMC dilution following supplier's recommendations. A bovine serum albumin (BSA) standard (Pierce) was serially diluted using PBS. Following 30 minutes incubation at 37°C, the optical density (OD) was measured at 570 nm.

### Flow cytometry

Antibodies used were as follows: anti-CD14 (BD Pharmingen: cloneM5E2), and anti-CD15 (Clone H198, BD Pharmingen). Anti-arginase I (HyCult Biotechnology: clone 6G3) and the isotype control (BD Pharmingen: clone MOPC21) were coupled with Alexa FluorR 488 (Molecular Probes). Cells were washed with PBS, the fixation step was performed with 2% formaldehyde in PBS and the permeabilization step with 0.5% saponin in PBS.

The determination of intracellular arginase was performed as described in [15]. The percentages for the isotype controls were <1.23%. Acquisition was performed using a FACSCalibur (BD Biosciences) and data were analyzed using Summit v4.3 software.

### Statistical analysis

Data were evaluated for statistical differences using a two-tailed Mann-Whitney test (GraphPad Prism 5) and differences were considered statistically significant at *p*<0.05. Unless otherwise specified, results are expressed as median±SEM.

## Results

### Clinical data

For this study, a cohort of 26 VL patients and 14 VL/HIV co-infected patients was recruited. All patients were male. The duration of illness was defined as the number of weeks since the patients noticed symptoms associated with visceral leishmaniasis: the most common symptom that they refer to was fever, but could also be weight loss, epistaxis, fatigue and abdominal swelling (as a sign of enlarged spleen or liver). For those patients with relapsing VL, the duration of illness only applies to the onset of the relapse. Duration of illness ranged from 2–24 weeks among the VL patients (4±1.2 weeks) and from 4–96 weeks among the VL/HIV patients (24±6.4 weeks)(data not illustrated). All patients had an enlarged spleen and liver, but the differences between the two groups were not significant (Table 1, *p*>0.05). Their liver and kidney functions were assessed, and as shown in Table 2, all the values were similar between VL and VL/HIV patients (p>0.05).

**Table 1:** Clinical data.

	VL patients	VL/HIV patients	*p* values
**Spleen size (cm)**	7.5±0.8	6.0±1.1	0.3086
**Liver size (cm)**	4.0±0.2	4.0±0.7	0.7379

Spleen and liver size = measurement below left costal margin and right subcostal margin (respectively).

**Table 2:** Liver and kidney functions assays.

	VL	VL/HIV	P values
**GOT (U/l)**	49.0±9.3	28.0±14.9	0.2033
**GPT (U/l)**	30.0±4.2	17.5±4.7	0.3505
**ALP (U/l)**	274.0±41.1	401.0±350.1	0.1900
**Creatinine (mg/dl)**	1.1±0.2	0.9±0.1	0.1495
**BUN (mg/dl)**	14.4±4.1	13.7±3.2	0.8582

GOT = glutamic-oxaloacetic transaminase, GPT = Glutamic-pyruvic transaminase, ALP = Alkaline phosphatase, BUN = blood urea nitrogen.

**Figure 1:**
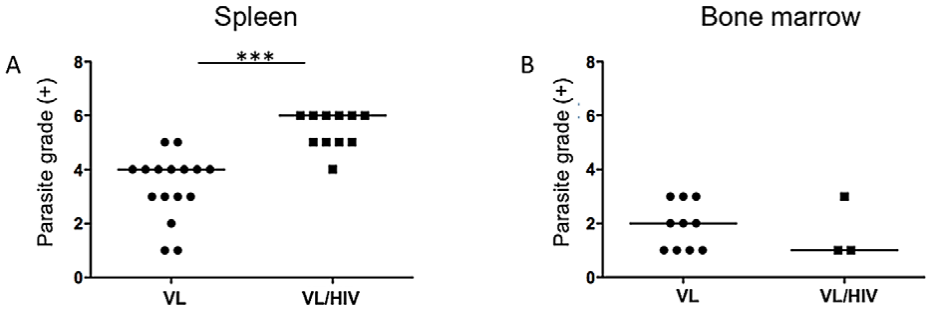
Parasite grade in spleen and bone marrow aspirates. The parasite grade was graded from 1+ to 6+ in smears from (A) spleen (VL patients: n = 16, VL/HIV patients: n = 11) and (B) bone marrow (VL patients: n = 10, VL/HIV patients: n = 3). The straight line represents the median.

The parasite grade was significantly higher in splenic aspirates from co-infected patients as compared to VL patients (6.0±0.3 vs 4.0±0.3, p<0.0001, Figure 1A). Parasite grade in the bone marrow aspirates appeared similar in both groups, however, it is difficult to draw a definite conclusion from these data, as there were only 3 values for the VL/HIV co-infected patients (Figure 1B).

**Figure 2:**
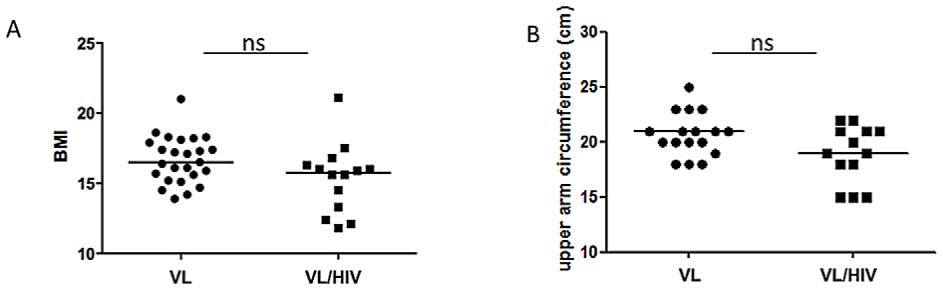
BMI and upper arm circumference. (A) Measurements of BMI (VL patients: n = 25, VL/HIV patients: n = 14) and (B) circumference of the upper arm (VL patients: n = 17, VL/HIV patients: n = 13). The straight line represents the median. NS = not significant.

The nutritional status of the two cohorts was assessed by calculating their body mass index (BMI) and measuring their upper arm circumference. As shown in Figure 2A, the medians BMI of both VL and VL/HIV groups were remarkably low and the median BMI of VL/HIV patients was similar to the BMI of VL patients (VL: 16.5±0.3 and VL/HIV: 15.8±0.6, *p* = 0.06). Twenty-three out of 25 VL patients and 13 out of 14 VL/HIV patients had a BMI<18.5; 12 out of 25 VL patients (48.0%) and 11 out of 14 VL/HIV patients (78.6%) had a BMI <16.5; indicative that the VL/HIV cohort was more severely malnourished. Although the median upper arm circumference was lower in the VL/HIV cohort than the VL cohort, this difference was not statistically significant (Figure 2B, 21.0±0.5 cm vs 19.0±0.7 cm, *p* = 0.1210).

**Figure 3:**
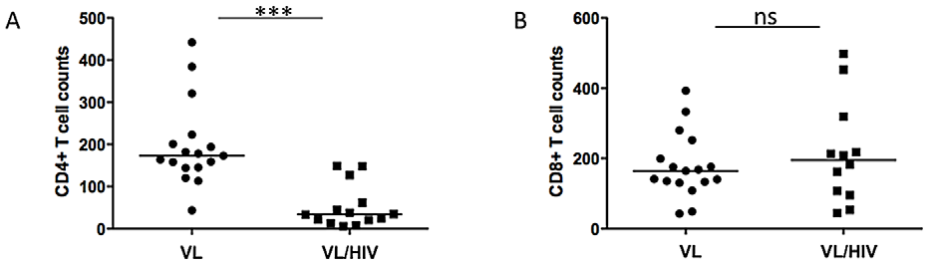
CD4^+^ and CD8^+^ T cell counts. (A) CD4^+^ (VL patients: n = 17, VL/HIV patients: n = 14) and (B) CD8^+^ (VL patients: n = 17, VL/HIV patients: n = 12) cell counts in the blood of controls were measured by flow cytometry. The straight line represents the median. Ethiopian national reference range for CD4^+^ T cells counts (cells/µl of blood) = 500–1300; Ethiopian national reference range for CD8^+^ T cells counts (cells/µl of blood) = 320–1800. NS = not significant.

Both CD4^+^ and CD8^+^ T cell counts were below the normal range in both groups of patients (Figures 3). Moreover, CD4^+^ T cell counts were significantly lower in VL/HIV patients (VL: 173±24.1, VL/HIV: 34.5±13.5, *p*<0.0001, Figure 3A). Although CD8^+^ T cell counts were below the normal range in both VL and co-infected patients (164±22.3 and 195±41.9 respectively, Figure 3B), the difference between the two groups was not statistically significant (*p* = 0.5799).

### Increased arginase activity in VL/HIV co-infection

**Figure 4:**
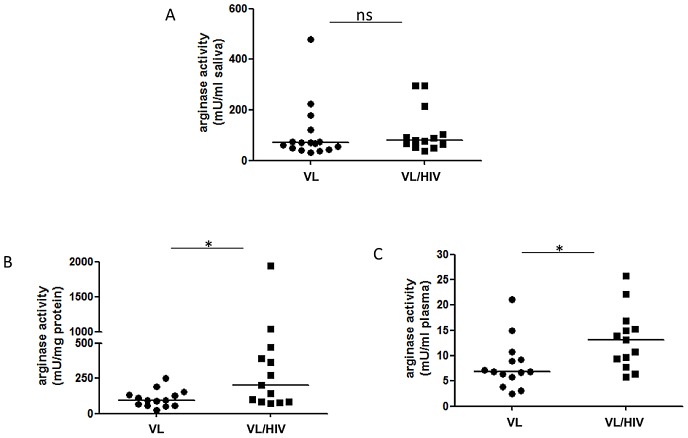
Arginase activity in saliva, PBMCs and plasma. The activity of arginase was measured by enzymatic assay in (A) Saliva (VL patients: n = 16, VL/HIV patients: n = 13), (B) PBMCs (VL patients: n = 14, VL/HIV patients: n = 13) and (C) plasma (VL patients: n = 14, VL/HIV patients: n = 13). The straight line represents the median. NS = not significant.

**Figure 5:**
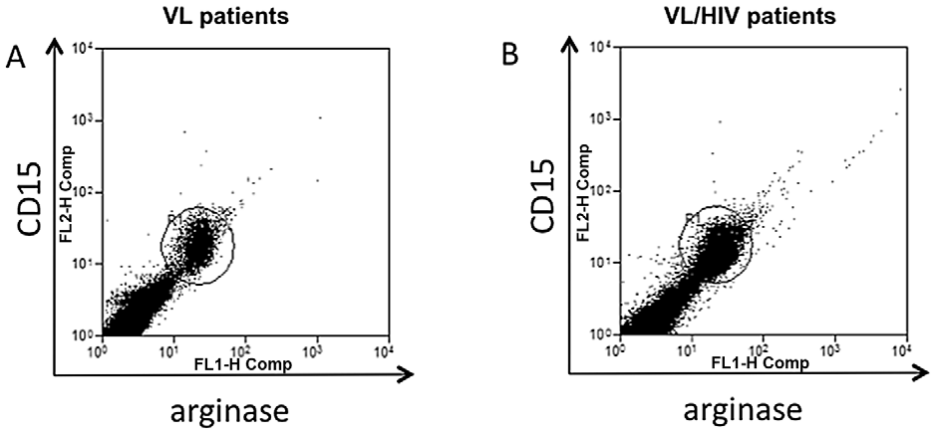
Phenotype of arginase expressing LDGs. PBMCs were isolated by density gradient from the blood of (A) one VL patient and (B) one VL/HIV patient and the phenotype of arginase-expressing cell was measured by flow cytometry. Data show the results of one representative experiment out of 8 independent experiments for the VL patients and 5 independent experiments for the VL/HIV patients.

Our previous study had shown that arginase activity was higher than normal in the blood of HIV+ patients [15] and in the blood of VL patients [16]. In the present study, we tested the hypothesis that arginase activity was even further increased in co-infected patients. In order to reduce the number of venipunctures, we tested whether arginase is detectable in saliva; our results show that it is clearly measurable in saliva (Figure 4A), however, the values obtained in each group were similar: VL: 70±28.2 mU/ml of saliva and VL/HIV: 80±25.1 mU/ml (*p* = 0.2925). Next we measured the levels of arginase activity in PBMCs and in plasma. The results in Figure 4B show that arginase activity was statistically significantly higher in PBMCs from co-infected patients (203±148.1 mU/mg protein) than those from VL patients (94.2±16.3, *p* = 0.0308). Similar results were obtained with the plasma: VL: 6.9±1.3 mU/ml vs VL/HIV: 13.1±1.6 mU/ml (p = 0.0162). These results show that arginase activity is even higher in PBMCs and plasma of co-infected patients as compared to VL patients. Finally, we determined the phenotype of arginase-expressing cells among the PBMCs. Our results are in agreement with our previous results [15], [16], [22] and show that neutrophils are the main type of cells in the PBMCs that express arginase. Indeed, in all 8 VL patients tested >98.9% of CD15+ and <0.24% were CD15− arginase+ (Figure 5A). Similarly, in all 5 VL/HIV patients tested, >98.2% of CD15^+^ cells expressed arginase and <0.31% are CD15^−^ arginase^+^ (Figure 5B). These results demonstrate that neutrophils are the main cells expressing arginase in the PBMCs of VL and VL/HIV patients.

## Discussion

The results of our study show that arginase levels are significantly higher in VL/HIV coinfected patients than in VL patients. We propose that increased arginase-mediated L-arginine metabolism contributes to T cell dysfunction and as a consequence, to the poor outcome associated with VL/HIV co-infection. We and others have shown that higher arginase activity coincides with lower levels of L-arginine and lower expression levels of CD3ζ in T cells [15], [16], [23], suggesting that T cell functions are compromised when activation occurs in the presence of suboptimal levels of L-arginine. One of the main immunological characteristics of both HIV and VL patients is the failure of their PBMCs to respond efficiently to antigen-specific restimulation [12], [13], [14], [24], [25]. This is even further impaired in VL/HIV co-infected patients: PBMCs from these patients proliferate less and produce considerably lower levels of IL-2 and IFN-γ in response to *Leishmania* and HIV antigenic restimulation [26]. And indeed, the CD4^+^ T cell counts, which are closely associated with disease severity and immune suppression [12], [27], [28], [29] were significantly lower in VL/HIV patients as compared to VL patients. The results presented in our study could explain this weakened immune response as we show that arginase, an enzyme with potent immunosuppressive properties, is even further increased in the PBMCs from VL/HIV patients. These higher levels of arginase activity may also explain why VL/HIV patients develop more frequent opportunistic infections, such as tuberculosis and pneumonia [5], [30].

In addition to impairing T cell functions, arginase activity has also been shown to favour parasite growth in macrophages: arginase catabolises L-arginine into polyamines, which are the main intracellular source of the polyamines necessary for the growth of the parasites [31]. Similarly, polyamines also appear be important for HIV replication, since blocking of a key enzyme in polyamine synthesis efficiently suppresses HIV-1 replication [32]. Therefore it is possible that polyamines resulting from the catabolism of L-arginine by arginase benefit both *Leishmania* and HIV replication. This might also account for the higher parasite grade in VL/HIV co-infected patients.

Arginase-expressing macrophages have been shown to promote parasite growth *in vitro* in experimental models of leishmaniasis [31], [33], and increased arginase activity at the site of pathology is a hallmark of nonhealing infections [31], [34]. However, the phenotype of arginase-expressing cells in the lesions has not yet been established. In human blood, the main arginase-expressing cell types are neutrophils [35], myeloid suppressor cells [36] and alternatively activated macrophages [37]. Here, we show that over 98% of CD15^+^ cells express arginase and only a minority of CD14^+^ monocytes express arginase. We and others have previously shown that a population of neutrophils co-purify with PBMCs following density gradient centrifugation [15], [37], [38], [39]. Because of their difference in density, these cells are referred to as low-density granulocytes (LDGs). Our data from HIV patients [20] and VL patients [16] suggest that these neutrophils have degranulated and that an increased frequency of LDGs coincides with disease severity: in HIV+ patients, we found a strong inverse correlation between the percentages of LDGs and CD4^+^ T cells counts [20], similar to that we found between arginase activity and CD4^+^ T cell counts [15]; in our VL study, we showed that the frequency of LDGs was significantly increased in PBMCs of patients before treatment.

Further characterization of these cells in VL/HIV patients will be essential to define how they contribute to exacerbated disease outcomes.

Severe malnutrition was remarkably common in VL/HIV patients: 78.6% of these patients had a BMI below 16.5. Malnutrition is associated with lower levels of L-arginine in the plasma [40], [41], [42] and we cannot exclude the possibility that malnutrition in these patients also contributed to the diminished levels of L-arginine in the plasma.

The spleen and liver sizes and the liver and kidney functions were similar in the VL and the VL/HIV patients, this is in agreement with the study by Hurisssa et al [1], that shows that the clinical presentation were similar in both groups of patients. In agreement too with the publication by Hurissa et al [1] was the intriguing finding that the duration of illness was longer in VL/HIV patients. The duration of illness was defined by the patient, from the onset of his symptoms, and it is therefore difficult to determine whether this information was accurate. However, it is possible that since T cells from co-infected patients display significantly more impaired immune functions [26], VL/HIV patients might therefore experienced symptoms earlier than the VL patients. It was also noticed that in contrast to VL patients, co-infected patients may not mount fever or only low grade fever, this would delay the process of seeking medical attention.

The development of new treatments that address the specific requirements associated with co-infection is of utmost importance. Our results have important implications for understanding the pathogenesis of VL/HIV co-infections. This may open up new therapeutic avenues to target dysregulated T cells responses. Further, the results of our study are not only applicable to understanding VL/HIV co-infections, but might also be relevant in understanding the exacerbated disease outcome associated with other co-infections.

## Supporting Information

Checklist S1 STROBE Checklist.
(DOC)

## References

[1] Hurissa Z, Gebre-Silassie S, Hailu W, Tefera T, Lalloo DG, et al (2010) Clinical characteristics and treatment outcome of patients with visceral leishmaniasis and HIV co-infection in northwest Ethiopia. Trop Med Int Health 15: 848–855. 20487426 10.1111/j.1365-3156.2010.02550.x

[2] (2006) Visceral leishmaniasis diagnosis & treatment guideline for health workers in Ethiopia. In: Ministry of Health E, editor. 1st Edition ed.

[3] Guerin PJ, Olliaro P, Sundar S, Boelaert M, Croft SL, et al (2002) Visceral leishmaniasis: current status of control, diagnosis, and treatment, and a proposed research and development agenda. Lancet Infect Dis 2: 494–501. 12150849 10.1016/s1473-3099(02)00347-x

[4] Ritmeijer K, ter Horst R, Chane S, Aderie EM, Piening T, et al (2011) Limited effectiveness of high-dose liposomal amphotericin B (AmBisome) for treatment of visceral leishmaniasis in an Ethiopian population with high HIV prevalence. Clin Infect Dis 53: e152–158. 22016502 10.1093/cid/cir674

[5] Alvar J, Aparicio P, Aseffa A, Den Boer M, Canavate C, et al (2008) The relationship between leishmaniasis and AIDS: the second 10 years. Clin Microbiol Rev 21: 334–359 table of contents. 18400800 10.1128/CMR.00061-07 PMC2292576

[6] Bernier R, Turco SJ, Olivier M, Tremblay M (1995) Activation of human immunodeficiency virus type 1 in monocytoid cells by the protozoan parasite Leishmania donovani. J Virol 69: 7282–7285. 7474154 10.1128/jvi.69.11.7282-7285.1995 PMC189654

[7] Zhao C, Papadopoulou B, Tremblay MJ (2004) Leishmania infantum enhances human immunodeficiency virus type-1 replication in primary human macrophages through a complex cytokine network. Clin Immunol 113: 81–88. 15380533 10.1016/j.clim.2004.06.003

[8] Mock DJ, Hollenbaugh JA, Daddacha W, Overstreet MG, Lazarski CA, et al (2012) Leishmania Induces Survival, Proliferation and Elevated Cellular dNTP Levels in Human Monocytes Promoting Acceleration of HIV Co-Infection. PLoS Pathog 8: e1002635. 22496656 10.1371/journal.ppat.1002635 PMC3320607

[9] Lodge R, Ouellet M, Barat C, Andreani G, Kumar P, et al (2012) HIV-1 promotes intake of Leishmania parasites by enhancing phosphatidylserine-mediated, CD91/LRP-1-dependent phagocytosis in human macrophages. PLoS ONE 7: e32761. 22412921 10.1371/journal.pone.0032761 PMC3295765

[10] Wolday D, Akuffo H, Fessahaye G, Valantine A, Britton S (1998) Live and killed human immunodeficiency virus type-1 increases the intracellular growth of Leishmania donovani in monocyte-derived cells. Scand J Infect Dis 30: 29–34. 9670355 10.1080/003655498750002268

[11] Bossolasco S, Gaiera G, Olchini D, Gulletta M, Martello L, et al (2003) Real-time PCR assay for clinical management of human immunodeficiency virus-infected patients with visceral leishmaniasis. J Clin Microbiol 41: 5080–5084. 14605142 10.1128/JCM.41.11.5080-5084.2003 PMC262523

[12] Clerici M, Stocks NI, Zajac RA, Boswell RN, Lucey DR, et al (1989) Detection of three distinct patterns of T helper cell dysfunction in asymptomatic, human immunodeficiency virus-seropositive patients. Independence of CD4+ cell numbers and clinical staging. J Clin Invest 84: 1892–1899. 2574188 10.1172/JCI114376 PMC304069

[13] Nylen S, Sacks D (2007) Interleukin-10 and the pathogenesis of human visceral leishmaniasis. Trends Immunol 28: 378–384. 17689290 10.1016/j.it.2007.07.004

[14] Goto H, Prianti MG (2009) Immunoactivation and immunopathogeny during active visceral leishmaniasis. Rev Inst Med Trop Sao Paulo 51: 241–246. 19893975 10.1590/s0036-46652009000500002

[15] Cloke T, Garvery L, Choi BS, Abebe T, Hailu A, et al (2010) Increased arginase activity correlates with disease severity in HIV seropositive patients. Journal of Infectious Diseases 202: 374–385. 20575659 10.1086/653736 PMC4663662

[16] Abebe T, Yegnasew T, Weldegebreal T, Cloke T, Closs E, et al. (Submitted for publication) Arginase: a marker of disease status in patients with visceral leishmaniasis. 10.1371/journal.pntd.0002134 PMC3610885 23556019

[17] Rodriguez PC, Ochoa AC (2008) Arginine regulation by myeloid derived suppressor cells and tolerance in cancer: mechanisms and therapeutic perspectives. Immunol Rev 222: 180–191. 18364002 10.1111/j.1600-065X.2008.00608.x PMC3546504

[18] Bronte V, Zanovello P (2005) Regulation of immune responses by L-arginine metabolism. Nat Rev Immunol 5: 641–654. 16056256 10.1038/nri1668

[19] Munder M (2009) Arginase: an emerging key player in the mammalian immune system. Br J Pharmacol 158: 638–651. 19764983 10.1111/j.1476-5381.2009.00291.x PMC2765586

[20] Cloke T, Munder M, Taylor GP, Müller I, Kropf P (submitted for publication) Characterization of a novel population of low-density granulocytes associated with disease severity in HIV seropositive patients. 10.1371/journal.pone.0048939 PMC3496742 23152825

[21] Chulay JD, Bryceson AD (1983) Quantitation of amastigotes of Leishmania donovani in smears of splenic aspirates from patients with visceral leishmaniasis. Am J Trop Med Hyg 32: 475–479. 6859397 10.4269/ajtmh.1983.32.475

[22] Abebe T, Hailu A, Woldeyes M, Mekonene W, Bilch K, et al (2012) Local increase of arginase activity in lesions of patients with cutaneous leishmaniasis in Ethiopia. PLoS NTD 6: e1684. 10.1371/journal.pntd.0001684 PMC3373636 22720104

[23] Zea AH, Culotta KS, Ali J, Mason C, Park HJ, et al (2006) Decreased Expression of CD3 zeta and Nuclear Transcription Factor kappa B in Patients with Pulmonary Tuberculosis: Potential Mechanisms and Reversibility with Treatment. J Infect Dis 194: 1385–1393. 17054067 10.1086/508200

[24] Munier ML, Kelleher AD (2007) Acutely dysregulated, chronically disabled by the enemy within: T-cell responses to HIV-1 infection. Immunol Cell Biol 85: 6–15. 17146463 10.1038/sj.icb.7100015

[25] Boasso A, Shearer GM, Chougnet C (2009) Immune dysregulation in human immunodeficiency virus infection: know it, fix it, prevent it? J Intern Med 265: 78–96. 19093962 10.1111/j.1365-2796.2008.02043.x PMC2903738

[26] Wolday D, Berhe N, Britton S, Akuffo H (2000) HIV-1 alters T helper cytokines, interleukin-12 and interleukin-18 responses to the protozoan parasite Leishmania donovani. AIDS 14: 921–929. 10853973 10.1097/00002030-200005260-00003

[27] Shearer GM, Clerici M (1991) Early T-helper cell defects in HIV infection. AIDS 5: 245–253. 1676274 10.1097/00002030-199103000-00001

[28] Levy JA (2007) HIV and the pathogenesis of AIDS; press A, editor.

[29] Appay V, Sauce D (2008) Immune activation and inflammation in HIV-1 infection: causes and consequences. J Pathol 214: 231–241. 18161758 10.1002/path.2276

[30] Russo R, Laguna F, Lopez-Velez R, Medrano FJ, Rosenthal E, et al (2003) Visceral leishmaniasis in those infected with HIV: clinical aspects and other opportunistic infections. Ann Trop Med Parasitol 97 Suppl 1: 99–105. 14678637 10.1179/000349803225002570

[31] Kropf P, Fuentes JM, Fahnrich E, Arpa L, Herath S, et al (2005) Arginase and polyamine synthesis are key factors in the regulation of experimental leishmaniasis in vivo. Faseb J 19: 1000–1002. 15811879 10.1096/fj.04-3416fje

[32] Schafer B, Hauber I, Bunk A, Heukeshoven J, Dusedau A, et al (2006) Inhibition of multidrug-resistant HIV-1 by interference with cellular S-adenosylmethionine decarboxylase activity. J Infect Dis 194: 740–750. 16941339 10.1086/507043

[33] Iniesta V, Gomez-Nieto, Corraliza I (2001) The inhibition of Arginase by Nw-Hydroxy-L-Arginine controls the growths of Leishmania inside macrophages. J Exp Med 193: 777–783. 11257143 10.1084/jem.193.6.777 PMC2193414

[34] Modolell M, Choi B-S, Ryan RO, Hancock M, Titus RG, et al (2009) Local suppression of T cell responses by arginase-induced L-arginine depletion in nonhealing leishmaniasis. PLoS Neglected Tropical Diseases 14: e480. 10.1371/journal.pntd.0000480 PMC2703824 19597544

[35] Munder M, Mollinedo F, Calafat J, Canchado J, Gil-Lamaignere C, et al (2005) Arginase I is constitutively expressed in human granulocytes and participates in fungicidal activity. Blood 105: 2549–2556. 15546957 10.1182/blood-2004-07-2521

[36] Rodriguez PC, Ernstoff MS, Hernandez C, Atkins M, Zabaleta J, et al (2009) Arginase I-producing myeloid-derived suppressor cells in renal cell carcinoma are a subpopulation of activated granulocytes. Cancer Res 69: 1553–1560. 19201693 10.1158/0008-5472.CAN-08-1921 PMC2900845

[37] Kropf P, Baud D, Marshall SE, Munder M, Mosley A, et al (2007) Arginase activity mediates reversible T cell hyporesponsiveness in human pregnancy. Eur J Immunol 37: 935–945. 17330821 10.1002/eji.200636542 PMC2699382

[38] Schmielau J, Finn OJ (2001) Activated granulocytes and granulocyte-derived hydrogen peroxide are the underlying mechanism of suppression of T-cell function in advanced cancer patients. Cancer Res 61: 4756–4760. 11406548

[39] Denny MF, Yalavarthi S, Zhao W, Thacker SG, Anderson M, et al (2010) A distinct subset of proinflammatory neutrophils isolated from patients with systemic lupus erythematosus induces vascular damage and synthesizes type I IFNs. J Immunol 184: 3284–3297. 20164424 10.4049/jimmunol.0902199 PMC2929645

[40] Padilla H, Sanchez A, Powell RN, Umezawa C, Swendseid ME, et al (1971) Plasma amino acids in children from Guadalajara with kwashiorkor. Am J Clin Nutr 24: 353–357. 5548742 10.1093/ajcn/24.3.353

[41] Poeze M, Bruins MJ, Luiking YC, Deutz NE (2010) Reduced caloric intake during endotoxemia reduces arginine availability and metabolism. Am J Clin Nutr 91: 992–1001. 20147469 10.3945/ajcn.2009.27812 PMC6443292

[42] Moyano D, Vilaseca MA, Artuch R, Lambruschini N (1998) Plasma amino acids in anorexia nervosa. Eur J Clin Nutr 52: 684–689. 9756126 10.1038/sj.ejcn.1600625

