# Correction: Arginase Activity in the Blood of Patients with Visceral Leishmaniasis and HIV Infection

**DOI:** 10.1371/annotation/9e89dee8-e2b7-4559-bd4e-933692403410

**Published:** 2013-01-25

**Authors:** Yegnasew Takele, Tamrat Abebe, Teklu Weldegebreal, Asrat Hailu, Workagegnehu Hailu, Zewdu Hurissa, Jemal Ali, Ermiyas Diro, Yifru Sisay, Tom Cloke, Manuel Modolell, Markus Munder, Fabienne Tacchini-Cottier, Ingrid Müller, Pascale Kropf

The affiliation for Dr. Tamrat Abebe was incomplete and should include an additional institution. The correct affiliation is as follows:

1. Department of Microbiology, Immunology and Parasitology , School of Medicine, Addis Ababa University, Addis Ababa, Ethiopia

2. Department of Biochemistry, WHO Immunology Research and Training Center, University of Lausanne, Lausanne, Switzerland

